# Miscellaneous Practical Items

**Published:** 1855-07

**Authors:** 


*Miscellaneous Practical Items:*

The following items we copy from the Virginia Medical and Surgical Journal:—Ed.

*Alopœcia Circumscripta.*—Mr. Startin, of London, has been lately treating baldness in a novel way. He first brushes over the spot with a solution of argent. nitr. (℈ij. ad ℥j.), which is followed, while moist, by one of the hydro sulph. of ammonia. A black sulphuret of silver is thus formed, which dyes the skin for a week or more. Whilst this application is evidently an irritant, it is more pleasant than the blistering fluid, cantharidine ointment and such remedies. At the same time the bald spot shows less when stained black, being nearer the color of the hair.

*Bronchitis—Chronic.*—The use of hydrochlorate of ammonia, in doses of fifteen or twenty grains, is highly spoken of by Dr. Delvaux (*Presse Med. Belge*) as a remedial agent in the treatment of chronic bronchitis. He precedes its administration with a purgative, and enjoins a strict diet during its continuance. Dr. Delvaux alleges that the cough will lessen and the dyspnœa become less, whilst the appetite improves. It causes an increased flow of urine, and also an augmentation of the cutaneous transpiration.

*Dysentery.*—The sedative influence of creosote has been appealed to by Dr. Wilmot, in the treatment of dysentery, with good results. He recommends this remedy as an enema of one drachm to twelve ounces of starch, and if we are to rely on his statements, there is something valuable in his suggestions.

*Enuresis.*—It is very well to have at hand various formulæ for this troublesome affection in children; and we select the following, which we owe to the Gazette De Hopitaux.

Dr. Blaschka, of Freyenwalde, uses equal parts of tinct. nucis vomica and tinct. ferri acet, of which 10 or fifteen drops should be taken twice each evening.

Dr. Huber, of Zurick, recommends ext. nucis. vom. 1 part, oxyd. ferr. niger. 48 parts, giving two grains night and morning.

Dr. Naegle gives one grain of tannin night and morning.

*Ganglion.*—To break what is commonly called a ganglion, and thus disperse the tumor which is often disfiguring the wrist, and about which we are often consulted, it is only necessary to flex the wrist so as to make the skin tense; then let the surgeon seize the hand with both of his and place both the thumbs, one above the other, on the ganglion. It is rarely that such pressure does not succeed in its object, whereas the usual way of placing the thumbs side by side, by the law of the diffusion of presence in fluids, the two counteract each other, and there is great loss of force.

*Glandular Enlargements.*—An ointment of black oxide of copper is thought of great value by Prof. Hoppe, of Basle, (*Deutshe Klinik,*) to discuss the various forms of glandular enlargements, so often occurring in practice. He has specially tested its virtues in indurations of the neck and of the salivary glands, goitres, and mammary enlargements. Prof. Hoppe believes that it would be equally applicable in orchitis, swelling of the groin, and in hypertrophied liver and spleen. It is prepared in the proportion of fifteen to thirty grains to the ounce of lard, and the parts to which it is applied should be covered with a bandage.

*Hemorrhoids.*—Guy's Hospital gives us the following ointment for piles, which happily combines the astringent properties of lead and galls, with an anodyne influence of opium. A good formulæ of this sort will be useful to the country practitioner, who will be frequently consulted with regard to this very common disease: ℞. Gallarum contrit., ʒij. ; Opii (emolliti aquæ cum, ʒj.) ʒss. ; Liq. plumbi. diacet., ʒij. ; Adipis, ℥j. ft. ung.

*Hydrocele.*—Prof. Langenbeck, of Berlin, not being satisfied with the effects of the iodine tincture as an injection in hydrocele, has recently been employing chloroform as a substitute, with the happiest results. He finds that it produces adhesive inflammation more quickly and more surely than the old remedy. After withdrawing the fluid of hydrocele, he injects about one drachm of chloroform, which remains for a short time, and then is allowed to escape. Langenbeck, in the *Deutshe Klinik*, reports four cases treated thus, which were radically cured in two or three weeks.

*Porrigo.*—This obstinate affection, often met with on the scalp in children, is attacked at Guy's Hospital with a prescription styled "Unguentum Metallorum," and prepared by mixing equal parts of zinc ointment, of the dilute nitrate of murcury, and of the cerate of acetate of lead. It has been very efficacious in porrigo, impetigo, and even in favus.

*Scarlatina.*—Sixty cases of scarlet fever, treated by Dr. Day, of Stafford, England, with nitric acid in full doses, has satisfied that gentleman that he has discovered an important remedy, and he reports his practice in a late number of the Medical Times and Gazette. There is but little difference in his idea of administering the nitric acid, which he uses also to throat as a gargle, and the old prescription of Fothergill, which he used to say "would pickle their juices,"—the muriatic acid diluted and sweetened with honey, which was employed internally and as a mouth wash. Others prefer lemonade draughts freely administered, which seems to have the same antiseptic virtue, and besides tend to the kidneys and skin. Dr. Day give the nitric acid in larger doses than we are in the habit of doing, and in this consists his only claim to originality.

*Skin Diseases.*—The active principle of hemlock is an oleaginous fluid called *conein*, and a Russian physician of some note, M. Murrawjeff, has found it efficacious, as an external application, in many of the obstinate diseases of the skin. It is very valuable in toothache, one drop being sufficient to control the pain. Murrawjeff, also, has tried it with advantage in neuralgic and syphilitic pains of the bones, and lastly, as a palliative in scrofulous and cancerous ulcer. Conein should be applied to the skin as an ointment, in the proportion of twelve drops to one ounce of cold cream. In neuralgia, Murrawjeff washes the part with wine, and applies two drops of pure conein, covering with oiled silk. One drop to two ounces of water forms a colyrium of great power; and as a enema, two or three drops may be administered in starch emulsion.—[Med Zeitung Russ.

*Spermatorrhœa.*—Professor Trosseau believes spermatorrhœa to depend on a spasmodic condition of the vesiculæ seminales, somewhat analogous to the condition of the bladder in enuresis.—He therefore proposes the use of belladonna, both internally, and applied as an ointment to the perineum nightly, and if necessary he advises a suppository of the extract of belladonna in the rectum. M. Trousseau declares that oftentimes an erotic feeling is conjoined to this spasm of the vesiculæ seminales, and suggests the use of bags of hot sand applied to the perineum at night. The use of bromide of potass, in doses of from 15 grs. to ʒss., as recommended by Thielman, will be found to have a decided anaphrodisiac effect.

*Syphilis—Secondary.*—M. Desmartis, of Bordeaux, declares that after a careful comparison of the effects produced by the different preparations of mercury, he has come to the conclusion that the cyanuret of mercury is of superior value, more especially in syphilis. He states that it never irritates or salivates, and where all the preparations of that metal had failed to produce benefit, that the cyanuret would restore to health, patients whose condition had seemed hopeless. M. Desmartis had also used it beneficially in iritis and syphilitic ulcerations of the nose and fauces.

